# Magnetic resonance imaging for diagnosis of suspected neurogenic thoracic outlet syndrome-a systematic scoping review

**DOI:** 10.3389/fphys.2023.1198165

**Published:** 2023-10-18

**Authors:** Pawel Szaro, Rohan Suresh, Brian Molokwu, Dhiraj Raju Sibala, Dhruv Mendiratta, Alice Chu, Aleksandra McGrath

**Affiliations:** ^1^ Department of Radiology, Institute of Clinical Sciences, Sahlgrenska Academy, University of Gothenburg, Gothenburg, Sweden; ^2^ Department of Musculoskeletal Radiology, Sahlgrenska University Hospital, Gothenburg, Sweden; ^3^ Department of Orthopaedics, Rutgers New Jersey Medical School, Newark, NJ, United States; ^4^ Department of Clinical Sciences, Umeå University, Umeå, Sweden; ^5^ Department of Surgical and Perioperative Sciences, Umeå University, Umeå, Sweden

**Keywords:** thoracic outlet syndrome, neurogenic, brachial plexus, magnetic resonance imaging, MRI, MRI protocol

## Abstract

**Background:** Neurogenic Thoracic Outlet Syndrome (nTOS) is a rare pathology caused by dynamic conditions or compression of neurovascular structures in the thoracic outlet region. nTOS can be difficult to diagnose due to nonspecific symptoms and magnetic resonance imaging (MRI) techniques are increasingly used to aid the diagnosis and surgical planning. This scoping systematic review explores how MRI is used for diagnosing nTOS and summarizes details of published MRI protocols.

**Methods:** A systematic screening of PubMed, Cochrane, Web of Science, and CINAHL databases using PRISMA-IPD guidelines was conducted in September 2022 to include full-text English papers on MRI and nTOS. Inclusion criteria involved studies describing MRI protocols for the diagnosis of TOS, with a focus on the imaging sequences and protocols.

**Results:** 6289 papers were screened to include 28 papers containing details of MRI protocols. The details of MRI protocols in the analyzed articles were incomplete in all studies. Most authors used 1.5T systems and included T1 and T2-weighted sequences. Most studies applied fat suppression, mainly with STIR. Positioning of the arm differed between studies, including neutral, hyperabducted and abducted and externally rotated positions.

**Conclusion:** Our review highlights a prevalent lack of detailed MRI protocol documentation for brachial plexus. Authors primarily rely on conventional 1.5T systems, employing standard T1 and T2-weighted sequences. The adoption of novel MRI sequences is notably lacking, and fat suppression techniques predominantly adhere to older methods as STIR. There is a clear imperative for authors to provide more comprehensive reporting of the MRI protocols utilized in their studies, ultimately enhancing comparability and clinical applicability. Establishing clear protocol reporting guidelines is crucial to allow for comparison between studies.

## 1 Introduction

Thoracic Outlet Syndrome (TOS) is a complex condition characterized by the compression of neurovascular structures either anatomically or dynamically in the thoracic outlet region. TOS is a rare pathology, estimated to be present in about 10 per 100,000 people, however it is likely to be more prevalent among athletes (Dengler et al., 2022). Neurogenic TOS (nTOS) is a subtype of TOS caused by compression of neurogenic structures contained within thoracic outlet. Compression of components of brachial plexus can cause a range of symptoms including pain, weakness, and numbness in the upper limb. This type of TOS can have a significant impact on the quality of life of affected individuals and can result in decreased work productivity and sport participation. nTOS is estimated to account for over 90% of all cases of TOS in adults (Illig et al., 2016; Jones et al., 2019), in contrast to being the cause of 38% of the total cases in the first two decades of life (Maru et al., 2009).

nTOS can be difficult to diagnose clinically due to its nonspecific symptoms and the close similarity of these symptoms to those of other conditions, such as peripheral neuropathy, cervical radiculopathy, and rotator cuff injuries. Furthermore, nTOS is a condition that can result from a variety of causes, including anatomical variations, repetitive motions, and postural habits. This makes it challenging for medical providers to make an accurate diagnosis of nTOS, especially when relying solely on a clinical examination. In the recent years there have been attempts to establish a clear and consistent set of diagnostic criteria for nTOS (Wade et al., 2019; Yeow et al., 2021), which are heavily dependent on combination of symptoms and positive clinical tests, however there is likely persistent and significant amount of variability in the diagnostic approach used by different medical providers.

In light of these challenges, the role of diagnostic imaging such as Magnetic Resonance Imaging (MRI) has been debated as an aid in the diagnosis of nTOS, similarly to other imaging modalities like ultrasound and neurophysiological studies. MRI is an expanding field where technological advances allow for increasing recognition of subtle morphological changes, with new synthetic techniques providing multiple tissue contrasts from a limited amount of MRI data (Kijowski and Fritz, 2023). High-field MRI systems allow for shortening the examination time and obtaining higher resolution in images and advances in adipose tissue suppression techniques make diagnosing nerve oedema easier and more precise (Casselman et al., 2022). The role of MRI in brachial plexus and nTOS imaging is not limited to diagnostics. By offering detailed images of the affected structures and providing valuable information for surgical treatment planning, MRI allows for evaluation of the brachial plexus and surrounding structures and visualization of anomalous anatomy, such as cervical ribs or bands (Jengojan et al., 2021). In the MRI imaging of traumatic brachial plexus injury, the systematic review of diagnostic accuracy of MRI for evaluating avulsion injuries found modest accuracy of MRI, with high bias in a third of included studies due to inadequate description of sequence descriptions (Wade et al., 2019). However, others report high diagnostic accuracy of MRI in identifying avulsions and presence of healthy nerve stumps for nerve reconstruction while providing detailed descriptions of protocols (Yeow et al., 2021).

The choice of the MRI protocol likely affects the possibility to identify discrete morphological differences in the brachial plexus and determines chances to correctly diagnose nTOS. This choice remains a subject of debate, with no framework or gold standard to guide the radiologists (Jengojan et al., 2022).

The aim of this scoping study is to systematically review MRI techniques applied for diagnosing nTOS, summarize existing MRI protocols including their advantages and limitations, and to identify knowledge gaps and recommend areas for further investigations.

## 2 Material and methods

This scoping review applied Arksey and O′Malley’s framework for scoping reviews (Arksey and O'Malley, 2005), as modified by Levac et al. (Levac et al., 2010). We adhered to the Preferred Reporting Items for Systematic reviews and Meta-Analyses Extension for Scoping Reviews (PRISMA-ScR) guidelines (Tricco et al., 2018).

### 2.1 Search strategy

The search strategy was developed by an iterative process with discussion among authors and consultation with information specialist (university librarian). Boolean searches were performed with terms appertaining to “Thoracic Outlet Syndrome” and “Magnetic Resonance Imaging”. PubMed, Cochrane, Cumulative Index to Nursing and Allied Health Literature (CINAHL) and Web of Science databases were searched between 9/10/2022 and 9/12/2022. No time limits were applied, and databases were searched for articles in English from inception.

### 2.2 Study selection

After the database searches were completed, duplicates were removed and the remaining articles were independently screened by four reviewers (RS, BM, DS, DM). The review team performed the title, abstract and full-text level selection according to the inclusion and exclusion criteria listed below. References of studies included at full-text level were crosschecked with the list of included studies. All disagreements within the review team were resolved by three of senior authors (AC, PS and AM).

Inclusion criteria involved studies describing MRI protocols for the diagnosis of nTOS, with a focus on the imaging sequences and protocols used. To identify nTOS in the published papers, we have applied the definition of American Society for Vascular Surgery (Dengler et al., 2022), who define nTOS by the presence of 3 of 4 criteria: pain and/or tenderness at the thoracic outlet, distal neurological changes, absence of other pathology, or positive response to scalene muscle injection.

The exclusion criteria included studies that do not focus on nTOS, for example, studies that describe arterial or venous TOS, studies that describe MR angiography as the primary imaging modality, studies which do not describe MRI protocols and studies focusing on cadaver subjects, where presence of nTOS diagnosis could not be confirmed. If the study stated clearly that the MRI protocols were applied for both vascular and nTOS, the study was included. Additionally, papers that were case reports, textbooks, letters to the editors, or studies that could not be used for extracting data were also excluded from the study.

### 2.3 Data extraction

Data extraction chart was drafted by PS and the process was performed similarly to study selection with two reviewers extracting data independently and any disagreements resolved in consultation with PS. Data extracted from each study included year of publication, type of article, number of patients, average age and age range, positioning of the arm during the exam, type of MRI system (strength of MRI field, 1,5T, 3T or higher), type of sequences (T1, T2, PD), type of fat suppression technique (STIR, DIXON, SPAIR, SPACE, FS), use of diffusion, if any, use of i. v. Contrast, use of 3D sequences, whether the MRI was bilateral or unilateral, plane of imaging (paracoronal or anatomical), gap, voxel size, and total scan time. The data extraction chart was devised based on data which would have to be provided in order to replicate the MRI protocol.

## 4 Results

**FIGURE 1:**
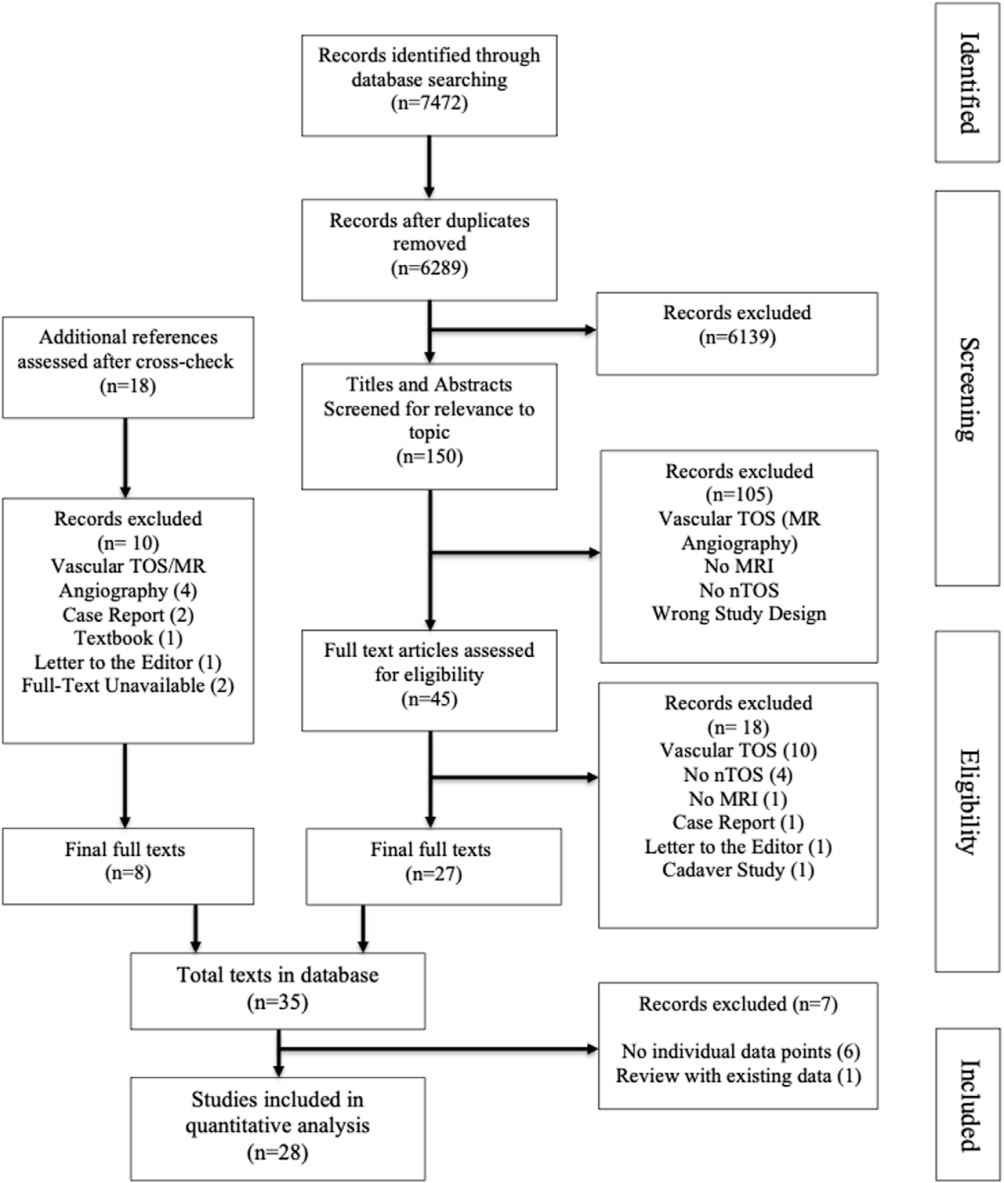
PRISMA flowchart depicting study selection process.

After removing duplicates, 6289 papers were screened at title and abstract level, with each entry screened independently by two reviewers, resulting in 150 full text papers. After fulltext review, cross checking references, and final round of excluding studies not describing MRI protocols, 28 studies were included in the database (see Figure 1, PRISMA flow chart, for details of inclusion and exclusion process). The included papers were published between 1987 and 2022. No bias assessment was performed and the parameters of MRI protocols for neurogenic TOS are summarized in a descriptive format, in keeping with the methodology of a scoping review.

Fifteen of 28 studies were review papers and 13 original studies. The studies highlight crucial aspects of diagnostic imaging for nTOS in both children and adults. They integrate clinical and radiological manifestation, comparing the value of MRI with functional assessments and ultrasound. In all studies, the details of MRI protocols were incomplete (see Table 1 for details of included studies) and did not allow for repeating the MRI examination by others.

**TABLE 1:** Summary of included studies.

Title of the study	Author	Year of publication	Study type
MR imaging findings in brachial plexopathy with thoracic outlet syndrome Aralasmak et al. (2010)	Aralasmak et al.	2010	Review
MRI findings in thoracic outlet syndrome Aralasmak et al. (2012)	Aralasmak et al.	2012	Original study
Thoracic outlet syndrome in 3T MR neurography—fibrous bands causing discernible lesions of the lower brachial plexus Baumer et al. (2014)	Baumer et al.	2014	Original study
MRI of thoracic outlet syndrome in children Chavhan et al. (2017)	Chavhan et al.	2017	Review
The relationship between magnetic resonance imaging findings and postural maneuver and physical examination tests in patients with thoracic outlet syndrome: results of a double-blind, controlled study Demirbag et al. (2007)	Demirbag et al.	2007	Original study
Thoracic Outlet: assessment with MR imaging in asymptomatic and symptomatic populations Demondion et al. (2003)	Demondion et al.	2003	Original study
Imaging assessment of Thoracic Outlet Syndrome Demondion et al. (2006)	Demondion et al.	2006	Review
Magnetic resonance imaging of traumatic and non-traumatic brachial plexopathies Fan et al. (2016)	Fan et al.	2016	Review
Thoracic Outlet Syndrome: diagnostic accuracy of MRI Hardy et al. (2019)	Hardy et al.	2019	Original study
High-resolution ultrasound and magnetic resonance imaging of abnormal ligaments in Thoracic Outlet Syndrome in a series of 16 cases Jengojan et al. (2022)	Jengojan et al.	2022	Original study
Imaging assessment of Thoracic Outlet Syndrome Khalilzadeh et al. (2021)	Khalilzadeh et al.	2021	Review
Clinical, electrodiagnostic and imaging features of true neurogenic Thoracic Outlet Syndrome: Experience at a tertiary referral center Kim et al. (2019)	Kim et al.	2019	Original study
Diagnosis of diseases of the supraclavicular region by use of MR imaging Kneeland et al. (1987)	Kneeland et al.	1987	Original study
Imaging of non-specific complaints of the arm, neck, and/or shoulder (CANS): role of the scalene muscles and piercing variants in neurogenic Thoracic Outlet Syndrome Koh (2021)	Koh	2021	Review
Choosing surgery for neurogenic TOS: the roles of physical exam, physical therapy, and imaging Kuwayama et al. (2017)	Kuwayama et al.	2017	Review
Pictorial essay: Role of magnetic resonance imaging in evaluation of brachial plexus pathologies Lawande et al. (2012)	Lawande et al.	2012	Review
Improved functional outcome in NTOS patients following resection of the subclavius muscle with radiological signs of nerve impingement: indication of participation of the subclavius in brachial plexus compression Liu et al. (2019)	Liu et al.	2019	Original study
Neurogenic thoracic outlet syndrome: current diagnostic criteria and advances in MRI diagnostics Magill et al. (2015)	Magill et al.	2015	Review
3 T MR tomography of the brachial plexus: Structural and microstructural evaluation Mallouhi et al. (2012)	Mallouhi et al.	2012	Review
ACR appropriateness criteria imaging in the diagnosis of Thoracic Outlet Syndrome Moriarty et al. (2015)	Moriarty et al.	2015	Review
Thoracic Outlet Syndromes and magnetic resonance imaging Panegyres et al. (1993)	Panegyres et al.	1993	Original study
Imaging of the Patient with Thoracic Outlet Syndrome Raptis et al. (2016)	Raptis et al.	2016	Review
Diagnostic value of magnetic resonance imaging in Thoracic Outlet Syndrome Singh et al. (2014)	Singh et al.	2014	Original study
Functional imaging of the thoracic outlet syndrome in an open MR scanner Smedby et al. (2000)	Smedby et al.	2000	Original study
MRI of brachial plexopathies Sureka et al. (2009)	Sureka et al.	2009	Review
MRI of the brachial plexus van Es (2001)	van Es	2001	Review
A novel approach for imaging of Thoracic Outlet Syndrome using contrast-enhanced magnetic resonance angiography (CE-MRA), short inversion time inversion recovery sampling perfection with application-optimized contrasts using different flip angle evolutions (T2-STIR-SPACE), and volumetric interpolated breath-hold examination (VIBE) Zhang et al. (2019)	Zhang et al.	2019	Original study
ACR Appropriateness Criteria^®^ Thoracic Outlet Syndrome Zurkiya et al. (2020)	Zurkiya et al.	2020	Review

When the strength of MRI field was considered, most authors used 1.5T systems (1.5T *versus* 3.0T n = 8 vs n = 7, one study reported low strength system of 0.5T) and included T1 (n = 19) and T2-weighted (n = 15) sequences in their protocols. No authors reported higher field systems (such as 7T scanners). PD images were not present in the analyzed articles. Most studies applied some form of fat suppression technique (n = 14), of which the STIR was dominant (n = 13), SPAIR was used in two studies and SPACE and DIXON in one. Bilateral imaging was reported only in some studies (n = 9).

The plane of imaging in the included studies varied. Imaging in both the plane of the course of the brachial plexus and the anatomical coronal plane was described in 11 studies. Ten articles reported imaging in the frontal anatomical plane and six in the plane of the brachial plexus. The use of 3D techniques was reported in 9 papers. When the information on the voxel size was provided (n = 6), the size ranged from 1.2 mm to 3 mm, and the gap ranged from 0.3 to 3.5 mm. The authors did not report the duration of the examination except for five studies, ranging from 8 to up to 60 min.

Positioning of the arm differed between studies and was frequently not documented in analyzed group. Majority of authors recommended examination in both neutral position (arms alongside the trunk) and hyperabducted position of the arm (n = 9), while others examined the patient only with arm in neutral position (n = 4) or hyperabducted (n = 3). In three studies, the patients were examined in neutral position and abducted and externally rotated arm (abduction and external rotation to 90°), while nine authors did not provide any details about positioning during the exam.

In the subgroup of original studies (n = 13), data regarding the sequences used were similarly incomplete. In 3 cases, there was no data on the strength of the MRI field used for the examination; in one case, low-field, 0.5T was used, and in the remaining cases, 1.5T (n = 5) and 3.0T (n = 3). Both T1 and T2-weighted images were used in 2 papers, T1 in 5 papers and T2 in 4 papers. None of the authors used PD sequences or diffusion imaging. Not all studies reported bilateral brachial plexus imaging, which was reported in only 4 studies. The fat suppression technique was used only in some cases (n = 5), most often STIR. Intravenous contrast was rarely used (n = 3). The frontal plane was oriented according to the course of the nerves in 2 studies, and in 3 cases, the anatomical plane was used; in 8 cases both methods were used simultaneously.

## 5 Discussion

The first conclusion from our study is that regardless of the type of the published studies, original studies *versus* reviews, the authors do not provide the complete brachial plexus MRI protocol, which prevents others from replicating the examination. There was no detailed description of which MRI sequences were used or what TE and TR are. There were no specifications in which planes the cross-sections were performed in and there was a lack of data on the gap and voxel size. In other words, the key parameters in imaging the brachial plexus were not provided. Another important parameter, which is duration of the examination, was rarely included. This is of high importance, as examination times are relevant to cost effectiveness and tolerability of the choice of this diagnostic method by patients.

While our study was a scoping review and did not involve a formal bias assessment, the lack of details of MRI protocols is an obstacle to performing any form of comparison of accuracy of MRI for diagnosing nTOS. Currently, there are no standards in either performing or reporting MRI protocols and findings for nTOS and TOS. Progress has been made however in providing both structural and even contextual templates (Mamlouk et al., 2018) for reporting of results of general MRI of brachial plexus. In contrast, in other areas such as fMRI an attempt has been made to provide general guidelines and a checklist for reporting methods, with a main goal of reproducibility of the examination (Poldrack et al., 2008). We argue that there is a need for achieving a similar consensus on reporting standards for brachial plexus MRI, ideally achieved by engaging key stakeholders, such as both musculoskeletal radiologists and brachial plexus surgeons.

Less than half of the original studies reported use of bilateral brachial imaging. Other authors suggest use of bilateral imaging for traumatic brachial plexus lesions (Hallinan et al., 2019), which greatly facilitates the capture of discrete signal changes of the brachial plexus that to even higher degree may occur in TOS pathology. Another controversial issue is value of imaging in hyperabducted position of the arm, which can reproduce symptoms of nTOS in this group of patients, (Dymarkowski et al., 1999; Lawande et al., 2012). Only less than half of included studies reported use of provocative positioning such as hyperabduction or combination of abduction and external rotation, which together with lack of other protocol details makes performing any assessment of effectiveness of such maneuvers impossible.

As the evidence was limited, the comparison of the value of different frontal planes was not possible. While there is no consensus on the most appropriate protocol for brachial plexus MRI, isovolumetric 3D sequences enabling reconstruction in different planes or multiplanar reformation (MPR) are beneficial, as they allow imaging of nerves traversing irregular planes (Szaro et al., 2022). The choice of paracoronal or coronal plane is thus likely to be of lower importance if 3D techniques were applied, such as when using isovolumetric voxels because the image can be reconstructed in any plane. Unfortunately, most authors do not report the voxel size in their research. In our opinion, the use of isovolumetric sequences facilitates nTOS imaging, similarly to traumatic brachial plexus protocols.

In recent years, there has been a dynamic development of new MR sequences that allow imaging of peripheral nerves. Unfortunately, our findings suggest that new sequences such as tractography or diffusion sequences were only used in one of included studies. Looking at the trends in those studies from 1987 onwards, there is a growing tendency to use newer methods of fat suppression. The STIR method is slowly being replaced by DIXON. DIXON method of fat suppression is seen as advantageous as it is designed to achieve uniform fat suppression, is less affected by artifacts than other techniques and increases conspicuity of imagined nerves without prolonging examination time (Wang et al., 2017). Even if choice of the most appropriate fat suppression technique can be challenging because of the region’s inhomogeneity, including the neck, superior thorax, and shoulders, these newer techniques of fat suppression are less susceptible to the inhomogeneity of the magnetic field around the brachial plexus.

For magnetic resonance imaging (MRI) of nTOS, sequences with contrast injection are often not required. However, contrast sequences can be helpful in the differentiation between tumors, infection, postoperative conditions, and post-radiation changes. On the other hand, the fluid sensitive sequences with maximal intensity projection (MIP) reconstruction show nerves as structures with a high signal and allow the radiologists to follow the nerves with relative ease (Kwee et al., 2014). The strength of the magnetic field is another factor which can have a significant impact on image quality and in the studies included in this review we have also noted a trend of increasing use of 3.0T over the years. The primary drawback of MRI in cases of nTOS stems from its static imaging nature. TOS is characterized by dynamic compression during movements like abduction, elevation, or retro-pulsion. Unfortunately, the size constraints of MRI tunnels significantly limit the range of arm positioning possible during imaging. This limitation may impede the accurate portrayal and evaluation of TOS’s dynamic aspects, potentially resulting in incomplete diagnostic insights.

The combination of limited protocol reporting and lack of prospective studies with appropriate design was seen in the literature about MRI and nTOS and the current state of the research field does not allow performing an effectiveness review. Investigating the diagnostic accuracy of MRI for nTOS retrospectively has shown low sensitivity and specificity (41% and 33%) (Singh et al., 2014). Others concluded that MRI has high specificity when providing guidance for planning of the surgical procedures is taken into account but lacks sufficient sensitivity as a screening test for diagnosis (Hardy et al., 2019). However, the comparison of MRI findings against the intraoperative findings only as used in both studies might be insufficient and better study design should include postsurgical patient reported outcome measures, as intraoperative findings might be subjective, surgeon dependent and prone to bias.

There is an ongoing discussion about whether diffusion tensor imaging will improve the accuracy of MRI of the brachial plexus. DTI has been evaluated experimentally for imaging of the brachial plexus in animal models of nerve injury (Chen et al., 2022) and in healthy volunteers (Oudeman et al., 2018) and as a proof of concept for avulsion injuries (Wader et al., 2020). Some authors report clinical use of diffusion images in their MRI protocol despite the artifacts (Vara et al., 2022). Only one study reported use of DTI specifically for nTOS and reported abnormalities in 30% of patients of those who also had morphological changes in standard 3.0 T MRI exam. DTI was found to be operator dependent, relatively time consuming and limited in the region of lower plexus due to breathing artifacts (Jengojan et al., 2022). As DTI is a relatively new modality, with the potential of detecting microstructural abnormalities and internal disorganization within the nerves, future development of examiner independent probabilistic tractography with lower motion sensitivity might lead to higher usefulness in diagnosing nTOS.

Limitations of this study are related to this review being a scoping study, as it does not include risk of bias or other quality assessment of included studies, uses a broad approach at the expense of depth and points to research that needs to be conducted. However, the scoping review method was appropriate as this research field is not ready for an effectiveness review.

MRI is a widely employed diagnostic imaging tool for evaluating the superior thoracic foramen, which includes the brachial plexus. Our study reveals a notable diversity in the MRI sequences employed to assess nTOS. Interestingly, studies seldom incorporated advanced nerve-specific sequences such as Diffusion Tensor Imaging (DTI), and infrequently utilized modern fat tissue suppression techniques. Given these findings, there emerges a clear imperative to establish standardized guidelines for MRI examination protocols in patients suspected of having nTOS. This standardization would optimize the exceptional capabilities of MRI in precisely assessing nerves and other soft tissues.

Diagnosing nTOS is a nuanced process that involves a comprehensive assessment. This includes a thorough clinical examination, functional evaluations employing nerve conduction examination, and a radiological investigation, often facilitated by MRI. MRI, being a imaging modality, offers detailed insights into the intricate anatomical structures relevant to nTOS. It enables precise localization of compression sites and assessment of nerve impingement severity. Depending on the specific case, supplementary imaging methods like ultrasound or computed tomography may be employed to complement the diagnostic process. These combined approaches equip clinicians with a comprehensive understanding of the condition, informing them in tailoring effective treatment plans.

A significant contradiction to undergoing an MRI is the presence of metallic implants or objects within the body. These can interfere with the magnetic field and potentially cause harm to the patient. Additionally, severe claustrophobia can be a contraindication, as the enclosed space of the MRI machine may cause distress.

The future research should focus on robust clinical studies with post-surgery outcome measures as comparators to clearly evaluate sensitivity and specificity of MRI, establishing clear protocol reporting guidelines which allow for comparison between studies and advancement of newer MRI sequences such as fat suppression and DTI.

## Data Availability

The original contributions presented in the study are included in the article, further inquiries can be directed to the corresponding author.
